# Molecular epidemiology of HIV-1 subtype B in the Basque Country (Spain)

**DOI:** 10.1186/1742-4690-9-S1-P33

**Published:** 2012-05-25

**Authors:** Juan Angel Patiño, Miguel M Thomson, Fernando González-Candelas

**Affiliations:** 1 Csisp-University of Valencia, Valencia, Spain

## 

The goal of this work was to study the HIV-1 subtype B epidemic in the Basque Country (Spain). For this, we used HIV samples submitted for genotypic testing of anti-retroviral resistance mutations from 2005 until 2008. Consequently, 2115 HIV-1 sequences comprising protease and retrotranscriptase (PR/RT) coding regions were analyzed. HIV transmission groups were identified by phylogenetic analysis. The 10 largest such groups were subsequently subjected to Bayesian phylogenetic and coalescent reconstructions, using a relaxed molecular clock model.

The results obtained show that these groups have been long-standing: most of them were originated in the late 70s or early 80s, and none after the year 2000. Most of these groups comprise both intravenous drug users (UDIs) and people who got infected through unprotected heterosexual sex (HTs). MSM clades were also represented in the sampled population. By comparing different demographic models, it was concluded that all the transmission groups are growing in an exponential manner. Time between infections was significantly lower in MSM groups than in those mainly containing IDUs (P-value < 0.05 in the Mann Whitney test).

In conclusion, this work suggests that in the near future the HIV-1 subtype B epidemics in the Basque Country will be characterized by a growth of the existing transmission groups. Due to the high diversity of these clusters, it is necessary to design campaigns for HIV prevention focused on the different risk groups.

